# Docetaxel and Gemcitabine Modulate Cellular Effects and Long Non-Coding RNA Profiles in Non-Small Cell Lung Cancer

**DOI:** 10.3390/ijms262311277

**Published:** 2025-11-21

**Authors:** Andrei-Alexandru Tirpe, Lajos Raduly, Oana Zanoaga, Stefan Strilciuc, Ioana Berindan-Neagoe

**Affiliations:** 1 Department of Genomics, MEDFUTURE Institute for Biomedical Research, Iuliu Hațieganu University of Medicine and Pharmacy, 400337 Cluj-Napoca, Romania; altirpe@gmail.com (A.-A.T.); lajos.raduly@umfcluj.ro (L.R.); oana.zanoaga@umfcluj.ro (O.Z.); strilciuc.stefan@umfcluj.ro (S.S.); 2 Doctoral School, Iuliu Hațieganu University of Medicine and Pharmacy, 400337 Cluj-Napoca, Romania; 3 The Academy of Medical Sciences, 030167 Bucharest, Romania

**Keywords:** gemcitabine, docetaxel, NSCLC, apoptosis, autophagy, MALAT1, NEAT1, HOTAIR, A549, CALU6, H520, H1703

## Abstract

Lung cancer remains the most prevalent and deadliest malignancy worldwide. According to the European Society for Medical Oncology guidelines for non-oncogene-addicted metastatic non-small-cell lung cancer (NSCLC), patients with metastatic squamous-cell carcinoma (LUSC) or metastatic non-squamous NSCLC with performance status 2 and PD-L1 < 50% may receive single-agent chemotherapy with gemcitabine (GEM), docetaxel (DOC), or vinorelbine. Herein, we investigated the cellular effects of GEM/DOC as monotherapies in NSCLC cell lines—lung adenocarcinoma, A549 and CALU6; LUSC, H520 and H1703. Treatment with GEM/DOC may induce apoptotic cell death in all NSCLC cell lines at 48 h. GEM/DOC can affect cancer cell migration assessed by scratch assay. Both GEM/DOC may produce distinct effects on cell cycle arrest, consistent with their particular pharmacodynamic effects. Furthermore, GEM/DOC induced signals consistent with autophagic activity in LUSC cell lines, but only GEM triggered signals consistent with autophagic activity in the CALU6 cell line. Analysis of three key long non-coding RNAs (lncRNAs)—MALAT1, NEAT1, and HOTAIR—showed variable expression in the studied cell lines as a potential response to DOC and GEM treatment. Our findings indicate different cellular effects of GEM and DOC in NSCLC cell lines and provide an overview of how currently used chemotherapeutics may influence the expression of lncRNAs.

## 1. Introduction

GLOBOCAN 2022 estimates report lung cancer as the leading malignancy in terms of incidence and mortality, with approximately 2.48 million new cases and 1.81 million deaths worldwide in 2022 [1]. Lung cancer is a complex malignancy in terms of molecular alterations, as diverse histological subtypes may harbor various somatic mutations [2]. From a histopathological standpoint, lung cancers are mainly categorized as small-cell lung cancers (SCLCs), accounting for approximately 15% of cases, and non-small-cell lung cancers (NSCLCs), comprising the other 85% of cases. NSCLC is further subclassified in lung adenocarcinoma (LUAD, approximately 40% of total cases), lung squamous-cell carcinoma (LUSC, approximately 25–30%), and large-cell carcinoma (LCC, approximately 5–10% of total cases) [3]. The present experiments will focus on NSCLC—specifically, LUAD and LUSC—as these are the most prevalent types of lung cancer. As a complex malignancy with an intricated tumor microenvironment [4], treatment strategies are varied and are dependent on numerous factors, including histopathological characteristics, disease stage, molecular attributes, and performance status. The approach to each case is personalized and may include surgical resection in resectable stages, as well as chemotherapy, immunotherapy, and targeted therapy, each with its own indications [5,6,7,8]. For patients with non-oncogene-addicted metastatic NSCLC (LUSC or non-LUSC), low PD-L1 expression, and performance status 2, cytotoxic chemotherapy—gemcitabine (GEM) or docetaxel (DOC)—is still a mainstay [7,8]. While these agents are known to enforce S-phase (GEM) or G2/M (DOC) checkpoints and trigger cell death [9,10], how they engage cell fate programs is heavily context-dependent.

Long non-coding RNAs (lncRNAs) are transcripts with over 200 nucleotides that have poor protein-coding abilities or do not possess protein-coding abilities [11]. However, through their role in regulating gene expression at the transcriptional and post-transcriptional level [12], lncRNAs are able to modulate various cancer processes, from angiogenesis (which is a common feature in NSCLC [13]) and epigenetic reprogramming to metastasis [11]. Studies showed that lncRNAs may also serve as a signature to treatment response [14]. LncRNAs are key modulators of apoptosis and autophagy. MALAT1, NEAT1, and HOTAIR integrate stress signals with chromatin, transcriptional, and post-transcriptional control, and have been separately linked to apoptosis and autophagy in several cancers [15,16,17,18,19]. We posited that the death phenotypes elicited by GEM/DOC in NSCLC are, at least in part, routed through these lncRNAs in a histology- and genotype-dependent manner.

In the present study, we profiled GEM and DOC as monotherapies across LUAD (A549, CALU6) and LUSC (H520, H1703) cell lines. Cellular outcomes included apoptosis (48 h), migration (scratch assay), cell cycle distribution, and autophagy readouts. In parallel, we quantified the expression of MALAT1, NEAT1, and HOTAIR and mapped them to canonical apoptotic/autophagy effectors to infer regulatory directionality.

## 2. Results

### 2.1. GEM and DOC Induce Cell Death in NSCLC Cell Lines at Specific IC_50_ Values

**Figure 1:**
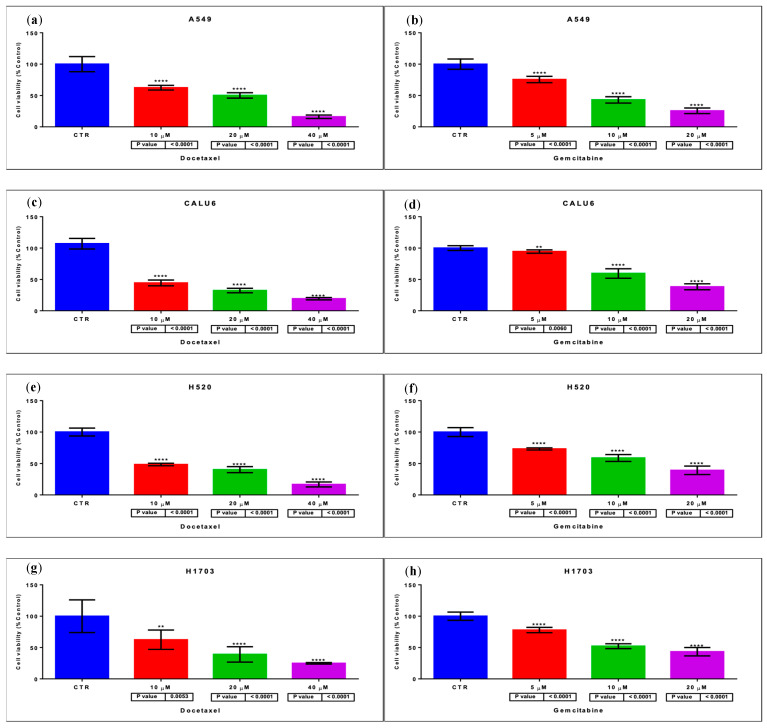
MTT assay for determining IC_50_ values for DOC and GEM for each cell line—(**a**) for DOC, A549; (**b**) for GEM, A549; (**c**) for DOC, CALU6; (**d**) for GEM, CALU6; (**e**) for DOC, H520; (**f**) for GEM, H520; (**g**) for DOC, H1703; (**h**) for GEM, H1703. two stars (**) mean *p* < 0.01 and four stars (****) denote *p* < 0.0001.

In order to determine the IC_50_ for GEM and DOC for each of the NSCLC cell lines—LUAD, A549 and CALU6, and LUSC, H520 and H1703—cells were treated with different GEM and different DOC concentrations in multiple MTT assay experiments, for 48 h. Final MTT assay readings showed that the approximate IC_50_ of GEM on the studied NSCLC cell lines was 10 µM (A549, *p* < 0.0001; CALU6, *p* < 0.0001; H520, *p* < 0.0001; H1703, *p* < 0.0001) and the IC_50_ of DOC on the studied NSCLC cell lines was 20 µM (A549, *p* < 0.0001; CALU6, *p* < 0.0001; H520, *p* < 0.0001; H1703, *p* = 0.0053)—Figure 1. Although the origin of the cell lines varied (e.g., A549 from male patient and CALU-6 from a female patient), the resulting concentrations were further used in the cellular and molecular experiments as the standard concentrations. The concentrations were also selected to enable consistent cross-line comparisons rather than identical inhibition levels.

### 2.2. Singular Treatment with GEM/DOC Induces Apoptosis in NSCLC Cell Lines

**Figure 2:**
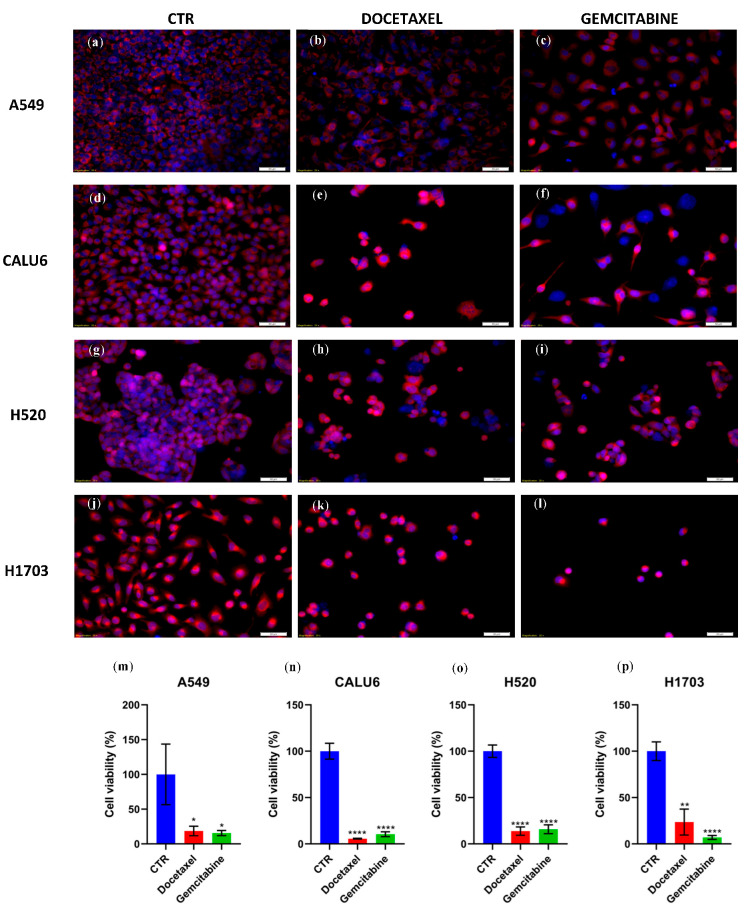
Cell viability via TMRE/Hoechst staining on fluorescence microscopy. TMRE is specific for mitochondrial membrane activity potential and Hoechst staining evaluates nucleus morphology and counting. (**a**) A549 cell line with no treatment; (**b**) A549 cell line treated with DOC 20 µM; (**c**) A549 cell line treated with GEM 10 µM; (**d**) CALU6 cell line with no treatment; (**e**) CALU6 cell line treated with DOC 20 µM; (**f**) CALU6 cell line treated with GEM 10 µM; (**g**) H520 cell line with no treatment; (**h**) H520 cell line treated with DOC 20 µM; (**i**) H520 cell line treated with GEM 10 µM; (**j**) H1703 cell line with no treatment; (**k**) H1703 cell line treated with DOC 20 µM; (**l**) H1703 cell line treated with GEM 10 µM. (**m**) Statistically, on the A549 cell line, treatment with DOC versus CTR showed statistical significance (*p* = 0.033), as well as GEM versus CTR (*p* = 0.0288), proving that these treatments decreased cell viability on the A549 cell line; (**n**) CALU6 cell line—DOC versus CTR (*p* < 0.0001) and GEM versus CTR (*p* < 0.0001), showing a statistically significant decrease in cell viability on fluorescence microscopy; (**o**) on the H520 cell line, DOC and GEM showed a statistically significant decrease in cell viability (*p* for both treatments < 0.0001); (**p**) on the H1703 cell line, both treatments decreased cell viability; however, GEM was statistically favored versus control with *p* < 0.0001; DOC versus control (*p* = 0.0015). One star (*) means *p* < 0.05, two stars (**) mean *p* < 0.01 and four stars (****) denote *p* < 0.0001. Scale bar: 50 µM.

Our results show that singular treatment with GEM/DOC induces apoptotic cell death in all NSCLC cell lines (A549, CALU6, H520, H1703) at 48 h of treatment, with a statistically significant decrease in cell population. The highest statistically significant decrease in the NSCLC cell population was induced by DOC and by GEM in the CALU6 cell line (*p* < 0.0001 for both treatments) and H520 cell line (*p* < 0.0001 for both treatments), as well as by GEM in the H1703 cell line (*p* < 0.0001). Figure 2 presents a comparison of untreated versus treated cell lines stained with TMRE/Hoechst and visualized on the Olympus IX71 (Olympus Corporation, Hachioji, Japan) inverted microscope.

**Figure 3:**
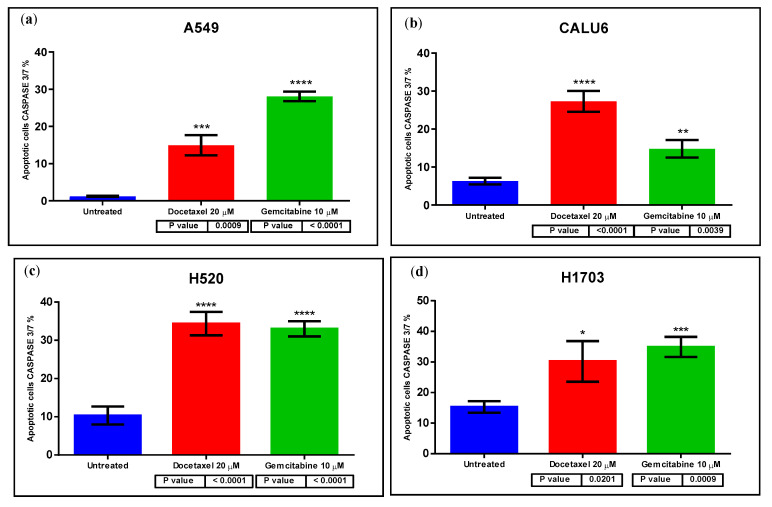
Apoptosis via caspase 3/7 on Celigo platform on cell lines (**a**) A549, (**b**) CALU6, (**c**) H520, and (**d**) H1703. All treatments induced significant apoptosis as detected via caspase 3/7 staining on Celigo platform. All treatments reached statistical significance on all cell lines. One star (*) means *p* < 0.05, two stars (**) mean *p* < 0.01, three stars (***) mean *p* < 0.001 and four stars (****) denote *p* < 0.0001.

Further apoptotic study with caspase 3/7 staining on the Celigo platform shows the highest statistically significant difference in apoptotic cells versus untreated cells in A549 cells treated with GEM (*p* < 0.0001), CALU6 cells treated with DOC (*p* < 0.0001), and H520 cells treated with DOC/GEM (*p* < 0.0001 for both individual treatments). Figure 3 presents these differences in apoptotic cells measured via caspase 3/7 staining on the Celigo platform.

### 2.3. GEM and DOC Alter Cancer Cell Migration in NSCLC Cell Lines

**Figure 4:**
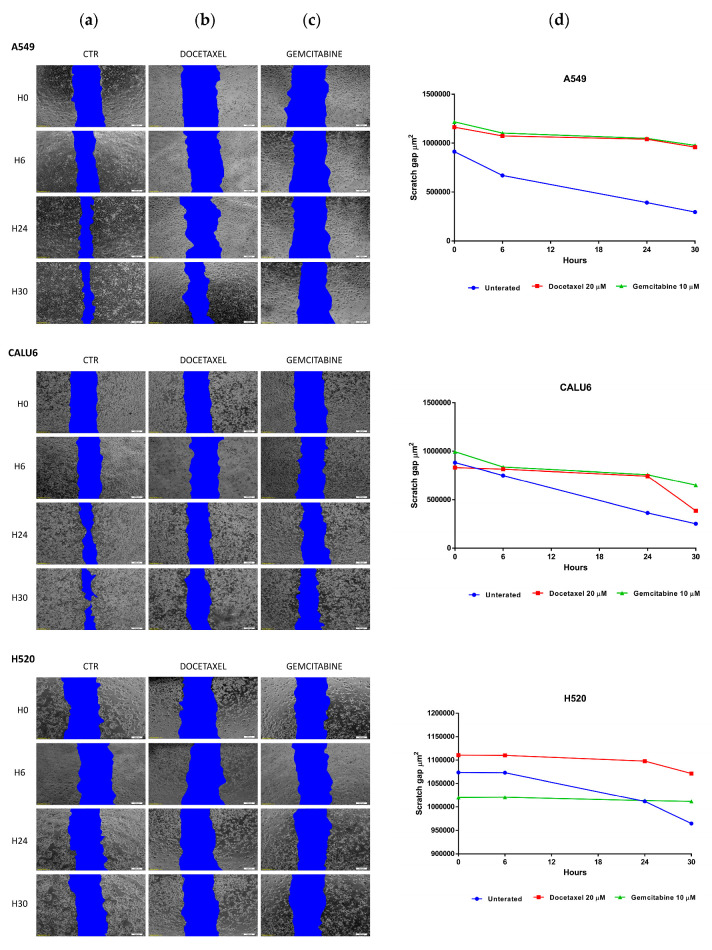

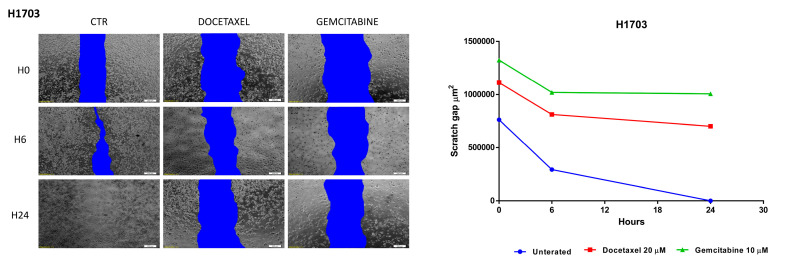
Scratch assay performed on each of the NSCLC cell lines taken into consideration—A549, CALU6, H520, and H1703. Potential migration was quantified by measuring the remaining wound area at 0, 6, 24, and 30 h. The variable healing rate of the control cell line can be observed in the (**a**) column, (**b**) represents the healing rate when treated with DOC and (**c**) represents the healing rate when treated with GEM. Concomitantly, column (**d**) plots the differential healing rate of each cell line (control versus treated cell lines).

To assess the migration capability of NSCLC cell lines A549, CALU6, H520, and H1703, we performed a scratch assay. Herein, we observed an increased capability of migration measured via the fastest healing rate of the untreated H1703 cell line. Globally, treatment with DOC and GEM produced a decrease in the healing rate capabilities of NSCLC cell lines A549, CALU6, H520, and H1703, as objectified by the present scratch assay (Figure 4).

### 2.4. Singular Treatment with GEM/DOC Induces Cell Cycle Arrest in Different Instances in NSCLC Cell Lines

**Figure 5:**
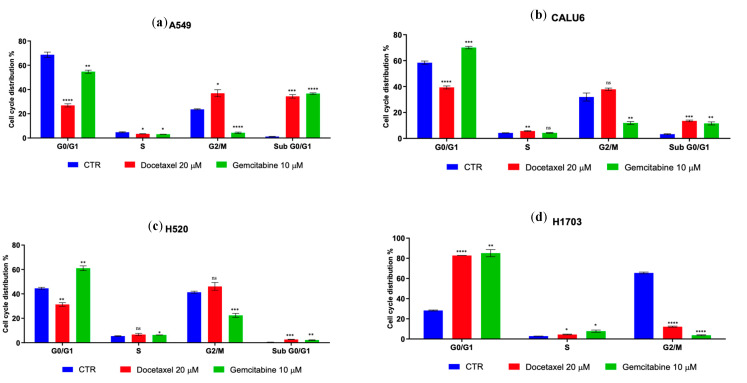
A comparison of cell cycle arrest of DOC/GEM treatments on each NSCLC cell line taken into consideration—LUAD (**a**) A549 and (**b**) CALU6, LUSC (**c**) H520 and (**d**) H1703. DOC is an anti-mitotic drug and thus stabilizes microtubules, while GEM is a nucleoside analog and interferes with DNA synthesis. ns = not significant, one star (*) means *p* < 0.05, two stars (**) mean *p* < 0.01, three stars (***) mean *p* < 0.001 and four stars (****) denote *p* < 0.0001.

To assess the effects of DOC and GEM on A549, CALU6, H520, and H1703 cell lines, we performed a cell cycle analysis on the Celigo platform, which showed varied results. First and foremost, consistent with its mechanism of action as a taxane/an anti-mitotic drug via microtubule stabilization, DOC increased the overall number of cells in G2/M versus the control (A549, CALU6) but decreased the number of cells in H1703 in G2/M; the effect on Sub-G0/G1 was also consistent with its mechanism of action, increasing the number of NSCLC cells in Sub-G0/G1 in all cell lines taken under consideration, except H1703. The overall effect on G0/G1 was a decrease in cell number, except in the H1703 cell line where the number of cells in G0/G1 was increased. When considering GEM, a nucleoside analog that interferes with DNA synthesis, the overall effect is consistent with the pharmacodynamic mechanism—an increase in number of cells/cell cycle arrest in the S phase and Sub-G0/G1 phase. The effect was not found to be statistically significant on the CALU6 cell line, whilst in A549, GEM had the opposite effect in the S phase. The variable efficacy of DOC and GEM in arresting cell cycle in different phases can be consulted in Figure 5. Table 1 presents the statistical data on each cell line on the status of treated cells versus the control.

### 2.5. Singular Treatment with GEM and DOC Induces Signals Consistent with Autophagic Activity in NSCLC Cell Lines

**Figure 6:**
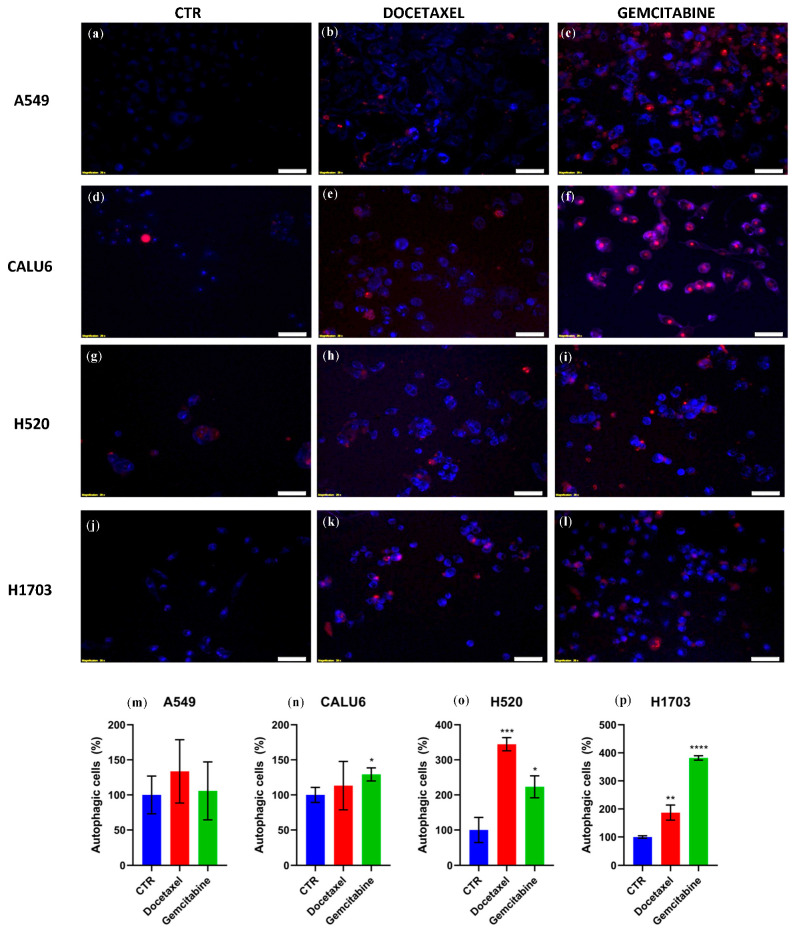
The autophagy assay was conducted via staining with MDC (Blue) for autophagic vacuoles, indicating autophagy activity and PI (Red) for nuclei, indicating compromised membrane integrity or cell death. (**a**) A549 cell line with no treatment; (**b**) A549 cell line treated with DOC 20 µM; (**c**) A549 cell line treated with GEM 10 µM; (**d**) CALU6 cell line with no treatment; (**e**) CALU6 cell line treated with DOC 20 µM; (**f**) CALU6 cell line treated with GEM 10 µM; (**g**) H520 cell line with no treatment; (**h**) H520 cell line treated with DOC 20 µM; (**i**) H520 cell line treated with GEM 10 µM; (**j**) H1703 cell line with no treatment; (**k**) H1703 cell line treated with DOC 20 µM; (**l**) H1703 cell line treated with GEM 10 µM. (**m**) There was no statistically significant difference between treated cells and the control in terms of autophagic cells in the A549 cell line; (**n**) in the CALU6 cell line, only GEM showed statistical significance in the percentage of autophagic cells (*p* = 0.0236), which was also evident in (**o**) the H520 cell line treated with DOC (*p* = 0.0005) or GEM (*p* = 0.0109), or in (**p**) the H1703 cell line treated with DOC (*p* = 0.0053) or GEM (*p* < 0.0001). one star (*) means *p* < 0.05, two stars (**) mean *p* < 0.01, three stars (***) mean *p* < 0.001and four stars (****) denote *p* < 0.0001. Scale bar: 50 µM.

In order to investigate the effect of singular treatment with GEM/DOC on autophagy, we performed an autophagy assay, using MDC (in blue) for staining autophagic vacuoles and PI (in red) for staining nuclei. In NSCLC cell lines A549, CALU6, H520, and H1703, treatment with DOC/GEM induced signals consistent with autophagic activity with variable effects. For the A549 cell line, there was no statistically significant difference between the rate of autophagic cells on treated cells versus the control. Conversely, there was a statistically significant difference—increase in autophagic cells—for GEM treatment on the CALU6 cell line (*p* = 0.0236) and for both treatments on the H520 cell line (DOC versus CTR, *p* = 0.0005 and for GEM vs. CTR, *p* = 0.0109), as well as on the H1703 cell line (DOC vs. CTR, *p* = 0.0053 and GEM vs. CTR, *p* < 0.0001). Figure 6 visually represents the effect of DOC and GEM on inducing signals consistent with autophagic activity in NSCLC cell lines.

### 2.6. Singular Treatment with GEM/DOC Induces LncRNA Expression Profile Alteration in NSCLC Cell Lines

**Figure 7:**
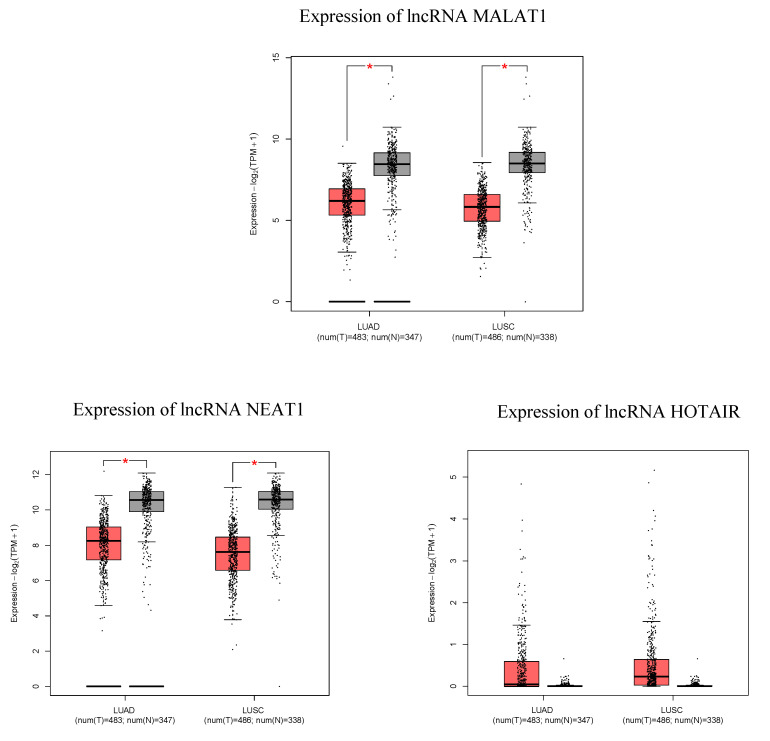
Bioinformatic analysis via the gene expression profiling interactive analysis 2 (GEPIA2, http://gepia2.cancer-pku.cn/, access date: 7 September 2025) tool [20] showing the differential expression of several lncRNAs that are implicated in NSCLC—lncRNAs HOTAIR, NEAT1, MALAT1, matched TCGA normal, and GTEx data comparatively for LUAD and LUSC. * statistically significant difference.

To further study the implication of GEM and DOC in treating LUAD and LUSC cell lines, we sought to identify molecular markers that were altered by these treatments (i.e., that are markers of response to treatment with DOC and GEM). As such, we screened for lncRNAs that were implicated in NSCLC development and progression and found lncRNAs MALAT1, NEAT1, and HOTAIR to be candidates for the subsequent experiments. Bioinformatic analysis on GEPIA2 tool [20] (Figure 7) confirms that these candidates are differentially expressed in LUAD/LUSC versus normal tissues; however, in the GEPIA2 analysis for HOTAIR, there was no statistically significant difference.

**Figure 8:**
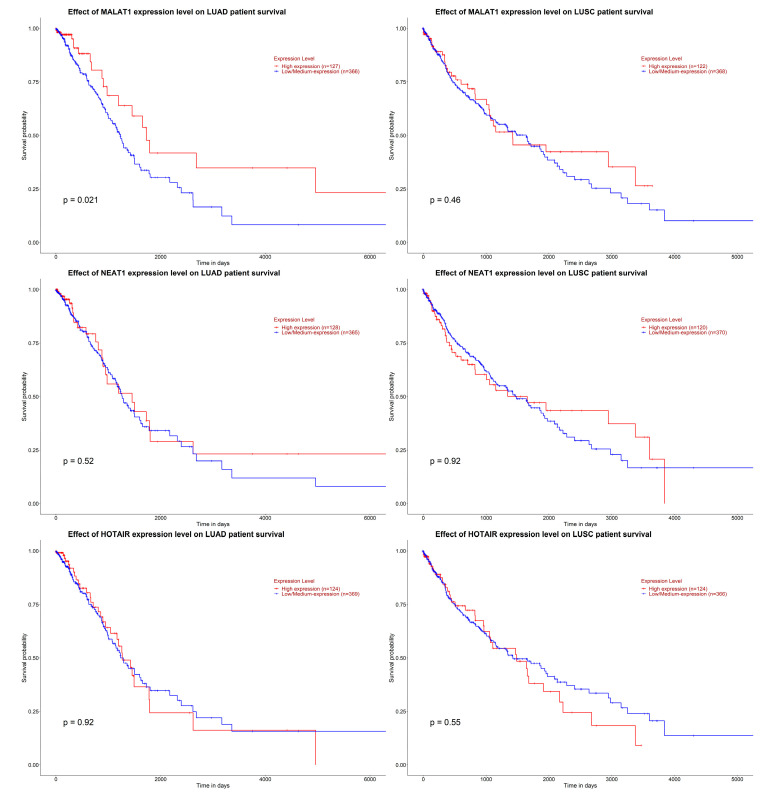
The effects of lncRNA MALAT1, NEAT1, and HOTAIR expression on patient survival in LUAD and LUSC. Survival analysis via the UALCAN tool (https://ualcan.path.uab.edu/, access date: 15 September 2025) [21,22].

Survival analysis via the UALCAN tool [21,22] showed that MALAT1 expression in LUAD was statistically significant (*p* = 0.021)—on the Kaplan–Meier survival curve, patients with high MALAT1 expression had a significantly improved survival probability. The other lncRNAs did not present statistical significance in relation to survival. This survival analysis can be consulted in Figure 8.

**Figure 9:**
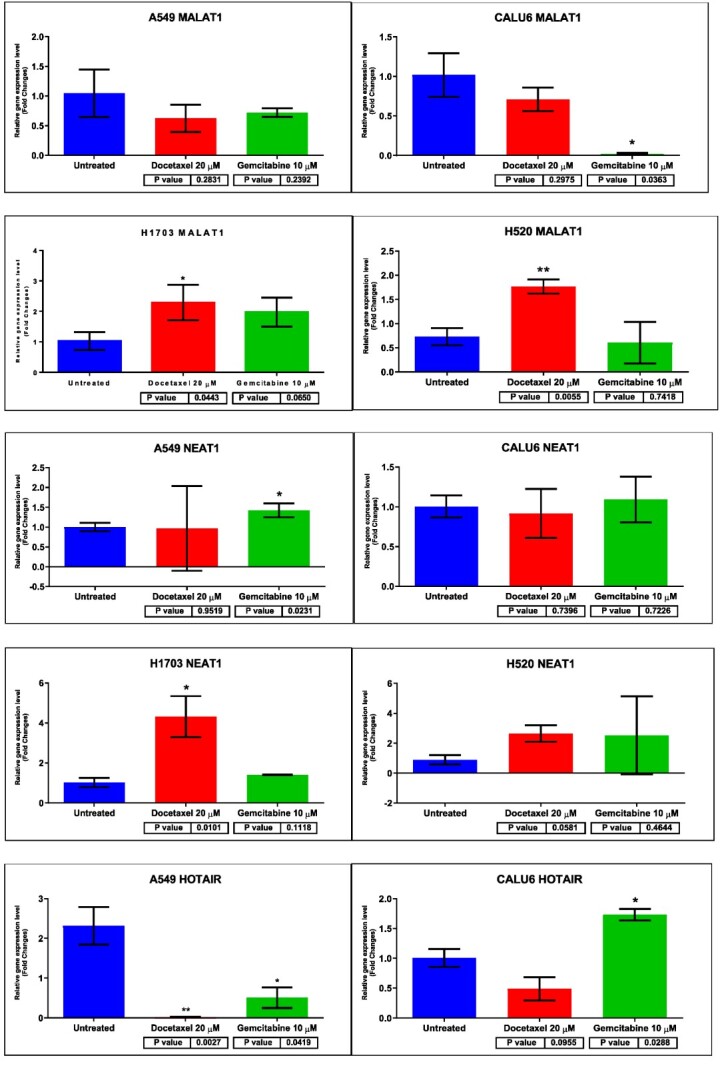

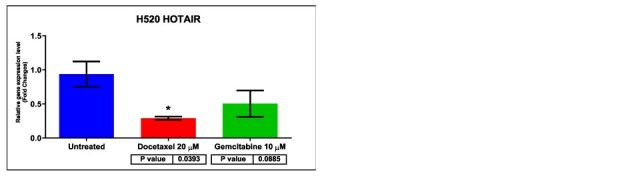
Differential expression of lncRNA MALAT1, NEAT1, and HOTAIR on NSCLC cell lines A549, CALU6, H520, and H1703 via qRT-PCR. The expression level of lncRNAs was compared between treated cell lines (with either DOC 20 µM or GEM 10 µM) and untreated cell lines, in order to identify alterations in lncRNA expression. One star (*) means *p* < 0.05 and two stars (**) mean *p* < 0.01.

Next, for each NSCLC cell line (A549, CALU6, H520, H1703), we employed quantitative reverse-transcription polymerase chain reaction (qRT-PCR) to identify differential expressions of the aforementioned panel of lncRNAs that were screened as potential candidates via bioinformatic analysis. We compared the expression of each lncRNA in the treated cell lines with untreated cell lines. Figure 9 provides a visual representation of the differences between these entities.

When considering lncRNA MALAT1, results varied. MALAT1 was found to be downregulated in the CALU6 cell line treated with GEM (*p* = 0.0363) and upregulated in LUSC cell lines H1703 and H520 treated with DOC (*p* = 0.0443 for H1703 and *p* = 0.0055 for H520). NEAT1 was upregulated in the A549 cell line treated with GEM (*p* = 0.0231) and in the H1703 cell line treated with DOC (*p* = 0.0101). Furthermore, lncRNA HOTAIR showed a general downregulated expression in the treated A549 cell line, with the highest statistical difference between DOC-treated cells and the control (*p* = 0.0027); the expression of HOTAIR on GEM-A549 treated cells was also downregulated (*p* = 0.0419) versus the control. On the CALU6 cell line, treatment with GEM led to a statistically significant upregulation of HOTAIR (*p* = 0.0288) versus the control. Moreover, on the H520 cell line, treatment with DOC led to a significant downregulation of HOTAIR lncRNA expression (*p* = 0.0393) versus the control. On the H1703 cell line, HOTAIR did not provide an adequate amplification. Although these data are correlative and not causal, they indicate a potentially significant association worthy of further investigation.

## 3. Discussion

Although newer therapies emerged in the past years in NSCLC, the use of DOC or GEM in monotherapy is still indicated in specific circumstances. According to the European Society for Medical Oncology (ESMO) guidelines, in a given case of non-oncogene-addicted metastatic NSCLC, single-agent chemotherapy (GEM, vinorelbine, DOC, pemetrexed, the latter in a non-squamous NSCLC setting) can be considered in patients with Eastern Cooperative Oncology Group (ECOG) performance status 2 and PD-L1 < 50% [7]. Furthermore, single-agent chemotherapy still remains the standard of care in elderly patients that are not eligible for doublet chemotherapy in this specific case [7]. In an open-label phase III randomized study, the DOC arm had significantly higher overall survival (OS) compared with the best supportive care (BSC) arm (*p* = 0.026) [23]. Furthermore, a phase III study (WJTOG 9904) compared DOC and vinorelbine in chemotherapy-naïve patients aged 70 years or older with NSCLC stage IIIB/IV with performance status 2 or lower. No statistically significant difference was found in the median OS between DOC and vinorelbine (14.3 months vs. 9.9 months, respectively), hazard ratio (HR) = 0.780 [95% CI: 0.561–1.085, *p* = 0.138). There was, however, a significant difference in terms of median progression-free survival (PFS) in the DOC arm vs. vinorelbine arm (5.5 months vs. 3.1 months, respectively; *p* < 0.001). Response rate (RR) was significantly higher in the DOC group vs. vinorelbine group (22.7% vs. 9.9%, *p* = 0.019) [24]. Another clinical trial studied the use of GEM monotherapy in elderly (>70 years), previously untreated patients that presented with stages IIIB-IV NSCLC. The overall RR was 22.2% [95% CI: 11.3–37.5], while the median time to disease progression was 5.1 months [95% CI: 3.5–6.7], the median OS was 6.75 months [95% CI: 5.3–8.2], and the median duration of response was 6.3 months [25]. It is essential to note that DOC and GEM have intrinsically different mechanisms of action. DOC is a potent semi-synthetic taxane that acts by inhibiting microtubule depolymerization, leading to cell cycle arrest in the G2/M phase and Bcl-2 phosphorylation with an ultimate effect of apoptotic cell death [9]. GEM is a pyrimidine nucleoside antimetabolite that is indicated in the treatment of several malignancies. When referring to the mechanism of action, deoxycytidine kinase activates GEM to dFdC-5′-monophosphate, leading to dFdC-5′-diphosphate and -triphosphate via further metabolization, with consecutive DNA integration and chain termination [10]. As a taxane drug, DOC modulates various cancer cell processes. In a study by He et al., DOC induced apoptotic cell death, inhibiting cancer cell proliferation in A549 and H460 cell lines [26]. GEM has various cellular effects in NSCLC. For instance, in a study by Pace et al., GEM induced apoptosis and presented antiproliferative effects [27]. Furthermore, in a study by Wu et al., both autophagy and apoptosis were induced by GEM in lung cancer cells A549 and SPC-A1 [28].

In the present study, we profiled GEM and DOC as monotherapies in NSCLC across LUAD cell lines (A549, CALU6), and LUSC cell lines (H520, H1703). Cellular outcomes included apoptosis, cancer-cell migration measured via scratch assay, cell-cycle distribution, as well as autophagy assay. In tandem, we quantified MALAT1, NEAT1, and HOTAIR expression via qRT-PCR and mapped the expression of these lncRNAs to canonical apoptotic/autophagy effectors to infer regulatory directionality.

**Cellular outcomes**. Herein, we have determined the final approximate IC_50_ value for DOC at 20 µM and for GEM at 10 µM, which were further considered as standard concentrations for the cell treatment process across the investigation. These concentrations were derived from several independent determinations, with minor variability between lines. These IC_50_ values were selected to enable consistent cross-line comparisons rather than identical inhibition levels. As expected, the IC_50_ values varied between studies. Table 2 presents a variety of studies that determined IC_50_ for DOC or GEM on NSCLC cell lines.

Furthermore, both treatments (GEM and DOC) induced apoptotic cell death in all NSCLC cell lines—A549, CALU6, H520, and H1703 at 48 h of treatment. We also performed a caspase 3/7 apoptotic study that supported this effect. Older studies showed that GEM was able to induce apoptosis in NSCLC on different cell lines—mucoepidermoid carcinoma, NCI-H292; LCC, NCI-CorL23; and adenocarcinoma, NCI-Colo699 [27]. In a study by Chen et al., combined treatment of MK-1775, a Wee1 inhibitor, with GEM, led to enhanced apoptotic effects in NSCLC cell lines A549, H460, HCC827, and H1975 [34]. In a study by Wang et al., combined treatment of DN604, a Pt(II) agent, with GEM effectively induced NSCLC cell apoptosis and reduced cancer cell motility [35]. Concomitantly, DOC induced dose-dependent apoptosis in A549 cells and H460 cells [26]. Furthermore, our study identified a potential overall effect of cancer-cell-migration inhibition under DOC/GEM treatment via non-mitomycin C-dependent scratch assay. We assessed the effect of DOC and GEM on the cell cycle via Celigo platform analysis. Treatment with DOC significantly decreased the number of cancer cells in the G0/G1 and S phases in the A549 cell line, suggesting a reduced number of cells entering early interphase and reduced DNA replication, respectively. The increase in cancer cell numbers in G2/M and Sub G0/G1 is consistent with DOC’s pharmacodynamic properties, acting as an anti-mitotic chemotherapeutic via inhibition of microtubule depolymerization. The same effect was observed on the other LUAD cell line, CALU6, for G0/G1 and G2/M, but not the S phase. However, on the H520 cell line, DOC failed to produce a statistically significant difference on the S and G2/M phases. Comparatively, in a study by Zhao et al., DOC induced cell cycle arrest in G2/M in A549 and NCI-H1299 [36]. GEM, a nucleoside analog, has a varying efficacy on the cell lines taken into consideration—for instance, GEM produces a statistically significant decrease in A549 cell populations in the G0/G1 phase, as well as S and G2/M phases, with an increase in Sub G0/G1. On the CALU6 cell line, GEM produces an increase in cell populations in the G0/G1 and Sub G0/G1 phases, with a decrease in G2/M. The autophagy assay showed that treatment with DOC/GEM induced signals consistent with autophagic activity with variable effects. GEM induced autophagic activity on CALU6 cell line with a statistically significant difference between treated cell lines versus control (*p* = 0.0236); concomitantly, both DOC and GEM induced autophagic activity on the H520 cell line with statistical significance between the treated cell line and control (*p* = 0.0005 for DOC-treated versus control; *p* = 0.0109 for GEM-treated versus control) and on the H1703 cell line (*p* = 0.0053 for DOC-treated versus control and *p* < 0.0001 for GEM-treated versus control). In a study by Pan et al., treatment with DOC on LUAD cells led to cytoprotective autophagy, protecting these cells from apoptosis [37]. Wu et al. found that GEM induced autophagy and apoptosis on lung cancer cell lines A549 and SPC-A1 [28]. It should be noted that the assessment of autophagy in this study was based on MDC/PI staining, which provided qualitative evidence of autophagic activity but did not allow distinction between autophagy induction and impaired autophagic flux. Therefore, our findings should be interpreted as signals consistent with autophagy rather than definitive proof of autophagy activation. Future studies employing LC3 and p62 quantification or flux inhibition assays are warranted to validate and extend these observations.

**LncRNA as regulators of cytotoxic stress**. LncRNA **MALAT1** is a key regulator in lung cancer pathogenesis and progression. Its expression can be induced by various stimuli, including hypoxia [38]. Hypoxia drives numerous cancer progression treats [39]. Studies have found MALAT1 to be implicated in resistance to cisplatin [40], migration and clonogenic growth [41], invasion [42] and other processes. In a study by Tiansheng et al., the authors found that MALAT1 was overexpressed in NSCLC cell lines and samples, and high MALAT1 expression correlated with worse histological grade, larger tumor size (defined as >3 cm), and metastasis [43]. Furthermore, in the Tiansheng study, MALAT1 had pro-oncogenic capabilities, as its overexpression enhanced NCI-H292 cell line proliferation rate and invasion abilities. The authors proposed a mechanism through the interaction between MALAT-1 and miR-202. Feng et al. showed that MALAT1 promotes cancer cell proliferation and resistance to gefitinib in lung cancer cells by sponging miR-200a, which is a regulator of ZEB1, further underscoring the key roles of MALAT1 in lung cancer [44]. In a study by Wang et al., MALAT1 was found to be implicated in cancer cell proliferation, migration/invasion, and inhibited apoptosis [15]. In our study, GEM may have induced statistically significant downregulation of MALAT1 in the CALU6 NSCLC cell line (*p* = 0.0363), whilst DOC may have induced statistically significant upregulation of MALAT1 in both LUSC cell lines H1703 and H520 (*p* = 0.0443 and *p* = 0.0055, respectively).

**NEAT1** is a potent and versatile lncRNA in NSCLC. In a study by Li et al., NEAT1 was significantly upregulated in NSCLC tissues compared with adjacent non-tumor tissues (*p* < 0.05), and high expression of NEAT1 was associated with advanced TNM stages, lymph node and distant metastasis (*p* < 0.05) [45]. The authors also showed that NEAT1 stimulated cancer cell proliferation and invasion in vitro. Mechanistically, Li et al. suggest that NEAT1 sponges miR-181a-5p, thus upregulating HMGB2 [45]. Chen et al. showed that NEAT1 modulated cancer cell proliferation, invasion and migration, as well as apoptosis via the hsa-miR-376b-3p/SULF1 axis in NSCLC [16]. In a study by Kong et al., hypoxia-induced HIF-2α increased NEAT1 expression and promoted epithelial to mesenchymal transition (EMT). The authors found a statistically significant correlation between high NEAT1 expression and several clinical parameters—including the TNM stage and metastasis. Kong et al. conclude that HIF-2α-induced NEAT1 upregulation in hypoxia may promote NSCLC progression via the miR-101-3p/SOX9/Wnt/β-catenin axis [46]. Furthermore, in a study by Sun et al., NEAT1 stimulated NSCLC cell growth in vitro, migration, and invasion, and had an inhibitory effect on NSCLC cell apoptosis [47]. In the present study, GEM may have induced NEAT1 overexpression compared to control in the A549 cell line (*p* = 0.0231), while DOC may have induced NEAT1 overexpression compared with the control in the H1703 cell line (*p* = 0.0101).

**HOTAIR** is a lncRNA with key roles in lung cancer, including resistance to cisplatin in LUAD cells [48], cell cycle control [49], metastasis [17] and others. In cancer, the development of chemoresistance leads to treatment failure, relapse, and even metastasis [50,51]. In a study by Li et al., HOTAIR was upregulated in gefitinib-resistant lung cancer cells, and its overexpression enhanced the malignant feature of gefitinib resistance [18]. The authors note that HOTAIR promotes cell cycle progression via EZH2/H3K27 and that this lncRNA is a key player in gefitinib resistance via EZH2, p16, and p21 regulation. In another study by Wang et al., HOTAIR was significantly downregulated in the PC9/R, H1975, H1299, and A549 cell lines, and in patients with primary/acquired EGFR-tyrosine kinase inhibitor (TKI) resistance. The authors found that high HOTAIR expression levels were significantly associated with longer PFS compared with low HOTAIR expression (*p* < 0.01), but in tumors that were responsive to EGFR-TKI therapy. Wang et al. show that in vitro overexpression of HOTAIR may restore chemosensitivity to gefitinib in gefitinib-resistant cell lines PC9/R, H1299, and A549 but not in H1975. Furthermore, HOTAIR overexpression activated EMT and led to PC9/R, H1299, and A549 apoptosis [52]. In our study, HOTAIR lncRNA expression was significantly downregulated versus the control, potentially via DOC and GEM treatment on the A549 cell line (*p* = 0.0027 for DOC and *p* = 0.0419 for GEM), whilst its expression was potentially upregulated by GEM treatment on the CALU6 cell line (*p* = 0.0288) versus the control. On the H520 cell line, treatment with DOC was associated with significantly decreased HOTAIR expression (*p* = 0.0393) versus the control.

Our findings indicate that GEM and DOC have different cellular effects in different NSCLC cell lines and provide an overview of how currently used chemotherapeutics may influence the expression of key lncRNAs in NSCLC—MALAT1, NEAT1, and HOTAIR.

The limitations of the present study include the in vitro characteristic, limited representation pertaining to the histology aspect (2 LUAD/2 LUSC cell lines) that do not entirely capture the genetic heterogeneity, limited mechanistic studies, limited lncRNA panel, and no validation in clinical samples/patient-derived study models. Bioinformatic datasets (UALCAN/GEPIA2-based) are heterogeneous and not directly matched to the in vitro setting and no direct correlation is implied. MDC/PI staining provides only qualitative evidence for autophagy.

## 4. Materials and Methods

### 4.1. Cell Lines and Cell Treatments

**Cell lines**

The present study considered two LUAD cell lines—A549 (derived from a male patient) and CALU6 (derived from a female patient, both from CLS Cell Lines Service GmbH, Eppelheim, Germany)-, and two LUSC cell lines—H520 (derived from a male patient) and H1703 (derived from a male patient, both from CLS Cell Lines Service GmbH).

**Treatments**

The chemotherapeutics taken into consideration were GEM (Thermo Scientific, Waltham, MA, USA) and DOC (Thermo Scientific, Waltham, MA, USA).

GEM is a pyrimidine analog that is widely used as a chemotherapeutic drug in the treatment of various cancers—from NSCLCs to breast cancers, ovarian cancers, bladder cancers, and others. GEM targets ribonucleotide reductase and interferes with DNA synthesis, leading to its therapeutic effect [53].

DOC is a potent taxane that is used in treating numerous cancers—from NSCLCs to gastric cancers and prostate cancers [54]. As a taxane, DOC interferes with microtubular dynamics via binding to ß-tubulin dimers, ultimately resulting in cell death [55].

**Figure 10:**
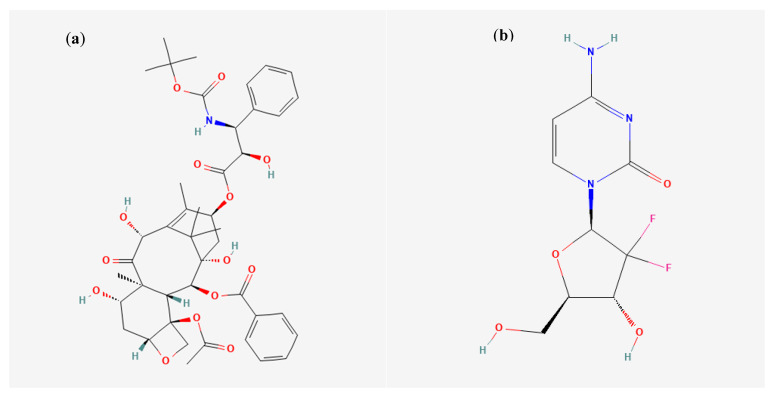
Molecular structures of (**a**) DOC [56] and (**b**) GEM [57]. Images retrieved PubChem @ National Library of Medicine.

Figure 10 presents the molecular structure of DOC and GEM.

In the present experiment, A549 cell line was cultured in DMEM-F12 supplemented with 10% FBS, 1% Glutamine, and 1% antibiotic (ZellShield, Minerva Biolabs GmbH, Berlin, Germany); CALU6 cell line was cultured in MEM supplemented with 10% FBS and 1% antibiotic (ZellShield, Minerva Biolabs GmbH, Berlin, Germany); H520 was cultured in RPMI, supplemented with 10% FBS, 1% Glutamine, and 1% antibiotic (ZellShield, Minerva Biolabs GmbH, Berlin, Germany); H1703 was cultured in RPMI, supplemented with 10% FBS, 1% Glutamine, and 1% antibiotic (ZellShield, Minerva Biolabs GmbH, Berlin, Germany). The culture media and supplements/antibiotics were acquired from Gibco^TM^ (Thermo Fisher Scientific, Grand Island, NY, USA). All cell lines (A549, CALU6, H520, H1703) were treated for 48 h with GEM 10 µM and DOC 20 µM individually, with no combination of GEM + DOC, as they are used as single-agent chemotherapy regimens. Next, various in vitro functional cellular assays were performed. Biological triplicates were used for all relevant experiments.

### 4.2. MTT Cell Viability Assay

From each cell line (A549, CALU6, H520, H1703), a number of 1 × 10^4^ cells/well were cultured in specific culture plates with 96-well structure for 24 h in 5% CO_2_ atmosphere at an incubation temperature of 37 °C, in biological triplicates. After the 24 h incubation time, all cell lines were treated in a systematical manner with GEM and DOC individually (for precision, multiple determinations were undertaken), at different concentrations of GEM (1 nM, 10 nM, 50 nM, 75 nM, 100 nM, 250 nM, 500 nM, 750 nM, 1 µM, 5 µM, 10 µM, 20 µM, 25 µM, 50 µM, 75 µM, 100 µM) and DOC (1 nM, 10 nM, 50 nM, 75 nM, 100 nM, 250 nM, 500 nM, 750 nM, 1 µM, 10 µM, 20 µM, 25 µM, 40 µM, 50 µM, 75 µM, 100 µM), for a higher precision in determining the IC_50_ dose. Next, 48 h post-treatment with GEM and DOC, the cell media were discarded and a well-determined 100 µL of 3-(4,5-dimethylthiazol-2-yl)-2,5-diphenyltetrazolium bromide (MTT) solution was added to each well of the plate, followed by 2 h of incubation at 37 °C. The MTT solution was then discarded, and the resulting formazan crystals were further solubilized by adding 100 µL of dimethyl sulfoxide (DMSO, Sigma-Aldrich). The absorbance was measured via the microplate reader Synergy H1 Hybrid Reader Biotek at 540 nm for the cell viability assay.

### 4.3. Cell Viability Assay

Fluorescence microscopy appraisal of apoptosis and cell viability was conducted using the Multi-Parameter Apoptosis Kit (Cayman, Ann Arbor, MI, USA) in biological triplicates. Cell staining with tetramethylrhodamin ethyl ester (TMRE) for mitochondria and Hoechst staining for cell nuclei was performed according to the manufacturer’s protocol. The stained cells were analyzed on the Olympus IX71 inverted microscope at UV wavelength 560/595 nm for TMRE and 350/461 nm for Hoechst. TMRE is specific for mitochondrial membrane activity potential and Hoechst staining evaluates nucleus morphology and counting. Furthermore, caspase 3/7 staining and Hoechst staining was used according to manufacturer’s protocol and apoptosis was evaluated for the GEM and DOC treatments using the Celigo Image Cytometer (Nexcelom, Lawrence, MA, USA) platform. *t*-test was used for further statistical analysis.

### 4.4. Cell Cycle Assay

In order to evaluate the cell cycle, all cell lines (A549, CALU6, H520, H1703) were cultured in specific culture plates with a 96-well architecture for 48 h, followed by treatment with GEM 10 µM and DOC 20 µM, individually, and incubation for 48 h with these treatments. Next, cells were fixed in ice cold methanol at a temperature of 4 °C for 30 min. After the fixation process, the cells were marked with propidium iodide (PI) solution and RNAse, and incubated in dark conditions at 37 °C in 5% CO_2_ atmosphere for 45 min. The cell cycle assay was performed on the Celigo Image Cytometer (Nexcelom) platform. Cell-cycle distribution was quantified automatically using the Celigo platform’s built-in analysis algorithm. Biological triplicates were used.

### 4.5. Autophagy Assay

Autophagy evaluation was executed using the Autophagy/Cytotoxicity Dual Staining Kit (Abcam, Cambridge, UK) according to the protocol. For this, staining with monodansylcadaverine (MDC) was performed for the staining of the autophagy vacuoles and propidium iodide (PI) for cellular death. After treatment in biological triplicates, cells were incubated for 48 h and were then visualized using the Olympus IX71 inverted microscope.

### 4.6. Scratch Assay

To evaluate cell migration, biological triplicates for cell lines A549, CALU6, H520, and H1703 were pre-treated individually in specific 24-well plates with GEM 10 µM and DOC 20 µM and then incubated for 48 h. Next, a scratch was created in each well by using a 20 µL pipette tip. Cells were then maintained in a medium with 1% serum. The resulting gaps were scaled (µm^2^) at hours 0, 6, 24, and 30 h and visualized via the Olympus IX71 inverted microscope.

### 4.7. RNA Extraction

Total RNA was extracted using TRIzol (TriReagent, Sigma-Aldrich, St. Louis, MO, USA) protocol from the untreated cell lines (A549, CALU6, H520, H1703) and from the individually treated cell lines with GEM 10 µM and DOC 20 µM.

### 4.8. DNAse Treatment

Total RNA samples were treated with DNAse in order to remove potential contamination with genomic DNA. Each 10 μL RNA sample was treated with 1.75 μL TURBO DNA-free mix according to manufacturer’s protocol. Next, the mixture was incubated at 37 °C for 30 min. After incubation, 2 μL of DNAse inhibitor was added in order to neutralize the activity of DNAse, followed by incubation at room temperature for 5 min. After this incubation, the samples were centrifuged for 2 min at 14,000 rpm. Next, the supernatant, which contained the purified RNA, was collected. The total RNA concentration was quantified using the NanoDrop technique to ensure the necessary purity and concentration for the subsequent qRT-PCR.

### 4.9. RNA Quantification: Nanodrop

The qualitative and quantitative evaluation was performed using the NanoDrop-1000 spectrophotometer (Thermo Scientific, Waltham, MA, USA).

### 4.10. Long Non-Coding RNAs Gene Expression Assessment via Polymerase Chain Reaction Assay

The lncRNAs that were evaluated via qRT-PCR were MALAT1, NEAT1, and HOTAIR. Housekeeping genes were B2M, GAPDH, and HPRT. Biological triplicates were used for qRT-PCR. The primer sequences used can be consulted in Table 3. After RNA extraction and RNA quantification, the synthesis of cDNA was performed according to the manufacturer’s recommendations using the High-Capacity Reverse-Transcription kit (Thermo Fisher Scientific, Waltham, MA, USA). Next, qRT-PCR was performed using the SYBR Select master mix (Applied Biosystems, Waltham, MA, USA) on the predefined qRT-PCR panel, according to the manufacturer’s protocol. Values for threshold cycle (Ct) were acquired via fluorescence emission. The normalization of Ct values was realized against the geometric mean of the three housekeeping genes. qRT-PCR data analysis was performed using the ΔΔCt method, based on fold-change calculations and consecutive normalization for all genes.

### 4.11. Statistical Analysis

The difference between the experimental conditions and control conditions were evaluated via standard deviation (mean ± standard deviation) and *t*-test; a *p* value of <0.05 was considered statistically significant. The statistical analyses and graphic representations were performed using the GraphPad Prism software version 10.6.1 and version 6 (GraphPad Software Inc., San Diego, CA, USA). For bioinformatic analysis of lncRNA expression level, TCGA data/TCGA normal-GTEx data were used via the gene expression profiling interactive analysis 2 (GEPIA2) tool on both LUAD and LUSC.

## 5. Conclusions

In conclusion, the present paper studied cellular and molecular effects of DOC and GEM treatments, as translated to single-agent chemotherapy, on NSCLC cell lines A549, CALU6, H1703, and H520. Our analysis shows that GEM and DOC produce robust cellular effects on treated NSCLC cell lines, ranging from apoptosis and autophagy induction to cell cycle arrest in specific phases, dependent on the drug mechanism of action. Herein, we also highlight the effect on cellular migration. These treatments positively affected cancer cells and led to significant changes in cell lines, with different magnitude of effects, proving their inhibitory effect upon lung cancer cells.

When considering the modulation of lncRNA expression, DOC and GEM treatment altered the expression levels of MALAT1, NEAT1, and HOTAIR in different cell lines, highlighting their potential use as markers of treatment response to treatment with DOC and GEM. DOC produced significant upregulation of MALAT1 in the H1703 and H520 cell lines, significant upregulation of NEAT1 in the H1703 cell line, and statistically significant downregulation of HOTAIR in the A549 and H520 cell lines. Conversely, GEM produced a significant downregulation of MALAT1 in the CALU6 cell line, significant upregulation of NEAT1 in the A549 cell line, and a significant downregulation of HOTAIR in the A549 cell line/upregulation of HOTAIR in the CALU6 cell line.

In conclusion, our findings indicate that GEM and DOC have different robust cellular effects in different NSCLC cell lines and provide a landscape of how currently used chemotherapeutics influence the expression of key lncRNAs in NSCLC—MALAT1, NEAT1, and HOTAIR.

**Perspectives**. As exemplified in this paper, chemotherapeutic drugs are able to influence the expression levels of key non-coding RNAs in NSCLC. In the present work, GEM and DOC induced the downregulation/upregulation of several lncRNAs in various NSCLC cell lines. Other newer therapies, such as immunotherapy/targeted therapy are currently being used successfully in NSCLC, with specific indications per the ESMO guidelines. It is abundantly clear that these therapeutics also have the potential to regulate the expression of non-coding RNAs. These alterations in non-coding RNA expression may serve as biomarkers for response, although further clinical studies are needed to introduce this into clinical practice.

## Figures and Tables

**Table 1:** Statistical analysis of cell cycle data—*p* values (*t*-test).

A549	G0/G1	S	G2/M	Sub-G0/G1
DOC vs. CTR	<0.0001	0.0233	0.0143	0.0006
GEM vs. CTR	0.0019	0.0159	<0.0001	<0.0001
**CALU6**				
DOC vs. CTR	<0.0001	0.0051	0.0691	0.0002
GEM vs. CTR	0.0003	0.9514	0.0038	0.0044
**H520**				
DOC vs. CTR	0.0010	0.1353	0.1137	0.0008
GEM vs. CTR	0.0017	0.0287	0.0003	0.0052
**H1703**				
DOC vs. CTR	<0.0001	0.0139	<0.0001	-
GEM vs. CTR	0.0011	0.0160	<0.0001	-

**Table 2:** DOC and GEM IC_50_ in various studies.

Treatment	Cell Line	Reported IC_50_	References
Docetaxel	A549	7.6 ± 1.8 nmol/L	[29]
	CALU-6	No publications identified for IC50 Docetaxel in CALU-6 cell line.	N/A
	H520	No publications identified for IC50 Docetaxel in H520 cell line.	N/A
	H1703	No publications identified for IC50 Docetaxel in H1703 cell line.	N/A
Gemcitabine	A549	391.2 ± 2.7 µM	[30]
		Varies from 84.2 µM to 26.7 µM (Gemcitabine-resistant cells with p21 knockdown that increased sensitivity to Gemcitabine)	[31]
		6.6 nM	[32]
		456.76 ± 37.62 nM	[33]
	CALU-6	No publications identified for IC50 Gemcitabine in CALU-6 cell line.	N/A
	H520	46.1 nM	[32]
	H1703	110.27 ± 3.62 nM	[33]

N/A = not applicable.

**Table 3:** Primer sequences for the qRT-PCR for lncRNAs and housekeeping genes.

Gene/LncRNA	Function	Primer Sequence
B2M	Housekeeping	FW-CACCCCCACTGAAAAAGATGAG RW-CCTCCATGATGCTGCTTACATG
GAPDH	Housekeeping	FW-AGAACATCATCCCTGCCTCTAC RW-CTGTTGAAGTCAGAGGAGACCA
HPRT1	Housekeeping	FW-TGACACTGGCAAAACAATGCA RW-GGTCCTTTTCACCAGCAAGCT
MALAT1	lncRNA	FW-TGTCCTTATAGGCTGGCCATT RW-AACTGCAGAGTTTGAGTGGTTTT
NEAT1	lncRNA	FW-GAGAAAAGTCCAAAAGGAGCAC RW-GGATGAGGCCTGGTCTTGT
HOTAIR-001	lncRNA	FW-GACAGGGTCTGGGACAGAAG RW-TCCAGGTTCCGGAAATCA

## Data Availability

The original contributions presented in this study are included in the article. Further inquiries can be directed to the corresponding author.

## Abbreviations

The following abbreviations are used in this manuscript:

ESMO	European Society for Medical Oncology
NSCLC	non-small-cell lung cancer
SCLC	small-cell lung cancer
GEM	gemcitabine
DOC	docetaxel
MALAT1	metastasis-associated lung adenocarcinoma transcript 1
NEAT1	nuclear-enriched abundant transcript 1
HOTAIR	HOX Transcript Antisense RNA
LCC	large-cell carcinoma
LUSC	lung squamous-cell carcinoma
LUAD	lung adenocarcinoma
LncRNA	long non-coding RNA
IC_50_	half-maximal inhibitory concentration
MTT	3-(4,5-dimethylthiazol-2-yl)-2,5-diphenyltetrazolium bromide
TMRE	tetramethylrhodamin ethyl ester
µM	micromolar
µm^2^	micrometer squared
CTR	control
H	hour
G0/G1 phase	gap 0/gap 1 phase
G2/M phase	gap 2/mitosis phase
S phase	synthesis phase
MDC	monodansylcadaverine
PI	propidium iodide
GEPIA2	gene expression profiling interactive analysis 2
UALCAN	University of Alabama at Birmingham cancer data analysis portal
qRT-PCR	quantitative reverse-transcription polymerase chain reaction
ECOG	Eastern Cooperative Oncology Group
PD-L1	programmed cell death-ligand 1
OS	overall survival
BSC	best supportive care
HR	hazard ratio
PFS	progression-free survival
RR	response rate
dFdC	difluorodeoxycytidine
DNA	deoxyribonucleic acid
Pt	platinum
ZEB1	zinc finger E-box-binding homeobox 1
TNM	tumor, node, metastasis staging system
miR	microRNA
HMGB2	high-mobility group box 2
EMT	epithelial to mesenchymal transition
HIF-2α	hypoxia-inducible factor 2 alpha
SOX9	SRY-Box Transcription Factor 9
EZH2	enhancer of zeste homolog 2
H3K27	histone H3 lysine 27
EGFR	epidermal growth factor receptor
TKI	tyrosine kinase inhibitor
DMEM-F12	Dulbecco’s modified eagle medium: nutrient mixture F-12
FBS	fetal bovine serum
MEM	minimum essential medium
RPMI	Roswell Park Memorial Institute medium
CO_2_	carbon dioxide
DMSO	dimethyl sulfoxide
